# Identification of Regions of Interest in Neuroimaging Data With Irregular Boundary Based on Semiparametric Transformation Models and Interval‐Censored Outcomes

**DOI:** 10.1002/sim.70309

**Published:** 2025-11-07

**Authors:** Chun Yin Lee, Haolun Shi, Da Ma, Mirza Faisal Beg, Jiguo Cao

**Affiliations:** ^1^ Department of Mathematics, Statistics and Insurance Hang Seng University of Hong Kong Hong Kong China; ^2^ Department of Statistics and Actuarial Science Simon Fraser University Burnaby Canada; ^3^ School of Medicine Wake Forest University Winston‐Salem USA; ^4^ School of Engineering Simon Fraser University Burnaby Canada

**Keywords:** Alzheimer's disease, EM algorithm, image analysis, survival analysis, triangulation

## Abstract

Alzheimer's disease (AD) is a progressive neurodegenerative disorder that leads to memory loss, cognitive decline, and behavioral changes, without a known cure. Neuroimages are often collected alongside the covariates at baseline to forecast the prognosis of the patients. Identifying regions of interest within the neuroimages associated with disease progression is thus of significant clinical importance. One major complication in such analysis is that the domain of the brain area in neuroimages is irregular. Another complication is that the time to AD is interval‐censored, as the event can only be observed between two revisit time points. To address these complications, we propose to model the imaging predictors via bivariate splines over triangulation and incorporate the imaging predictors in a flexible class of semiparametric transformation models. The regions of interest can then be identified by maximizing a penalized likelihood. A computationally efficient expectation–maximization algorithm is devised for parameter estimation. An extensive simulation study is conducted to evaluate the finite‐sample performance of the proposed method. An illustration with the AD Neuroimaging Initiative dataset is provided.

## 1 Introduction

Alzheimer's disease (AD) is a condition that affects a large number of individuals globally. A report from Alzheimer's Disease International in 2018 indicated that over 50 million people globally are currently living with dementia, a number projected to triple by 2050. In the meantime, structural magnetic resonance imaging (MRI) has been widely used to gain insights into the underlying processes of AD in individual patients. Identifying the brain regions linked to the disease progression from MRI scans has become an area of crucial research interest, essential for developing targeted treatments and interventions.

Numerous retrospective studies have investigated patients with AD to explore how different brain regions contribute to its onset and advancement. The hippocampus is widely recognized as the key region associated with early‐stage disease development due to its involvement in forming long‐term memories. Additionally, given the network of interconnected brain regions, it is likely that the onset and progression of AD may involve regions beyond the hippocampus. For example, the entorhinal cortex has been shown to associate with the preclinical stage of AD progression, whose posterior‐lateral subfields are most susceptible to early neurofibrillary tangle [1]. Presubiculum and subiculum subfields have been found to exhibit the earliest atrophy and are predictive of memory performance in the progression of the disease [2]. Recent studies have also shown from a gradient pattern of pathological markers that the amygdala serves as a hub for propagating AD pathology through its connections with other brain regions [3]. Other key regions related to the disease progression include, for example, ventricle, basal forebrain, cingulate cortex, and white matter tracts [4, 5, 6, 7].

Among the brain regions considered in various retrospective studies, to reliably identify the most crucial one that is *statistically* associated with the progression of AD, we propose a data‐driven approach for the survival analysis of the large MRI datasets from the Alzheimer's Disease Neuroimaging Initiative (ADNI) study. Such a method can help corroborate or challenge existing hypotheses about the role of the various brain regions in the disease progression and may find patterns that may not be apparent through traditional retrospective research methods.

Our objective is to develop a reliable model for detecting regions of interest within brain MRI scans that are associated with the onset of AD. There exist a few statistical challenges to this objective. First, in the ADNI study, the regularly followed‐up patients were clinically assessed only at each revisit time point to ascertain the onset or progression of the disease. Therefore, the exact time for the conversion of AD was unknown but only observed to fall between two revisit time points, hence interval‐censored [8, 9, 10]. The standard estimation procedure, such as the partial likelihood, cannot be directly applied to interval‐censored data. Second, since the brain area within MRI scans is confined within an irregular region with noise elsewhere, using existing methods for rectangular (regular) boundaries on such images would result in boundary leakage issues [11]. A direct consequence of the boundary leakage is that the background information would be incorrectly included in the analysis due to the dominance of sharp transitions between the image and background, as demonstrated in several examples in Ramsay [12].

To address these challenges, we propose a novel model for identifying key areas in brain MRI scans that are statistically associated with the progression of AD. Our method specifically addresses the difficulties related to the irregular contours found in MRI images and the interval‐censored data based on a flexible class of semiparametric transformation models. We propose to approximate the image with irregular boundaries via bivariate polynomial splines constructed on triangulation grids as proposed in Lai and Schumaker [13] and Lai and Wang [14]. By utilizing piecewise polynomial functions on a triangulated surface, the brain scans can be smoothly incorporated into a survival model. This allows for the precise location of the area of interest within a regression framework. Moreover, to incorporate the interval‐censored data, we develop a Poisson data augmentation approach, which is shown to be computationally efficient and powered by the Expectation Maximization (EM) algorithm. Finally, as some but not all regions of the brain are of interest, we propose to incorporate group penalty terms on the imaging predictors, selectively emphasizing areas implicated in disease onset. The regions of interest can then be identified by maximizing a penalized likelihood. The R codes for implementing our method in this paper are publicly available on GitHub: https://github.com/lcyjames/Roiico.

Incorporating high‐dimensional images for survival models has received considerable attention recently, with a majority of the research work focused on functional principal component analysis (FPCA). Zipunnikov et al. [15] established a linkage between the FPCA and singular value decomposition, which allows for computationally efficient estimation of the single‐level FPCA for vectorized images. To retain the spatial structure of the image, Reiss and Ogden [16] studied the functional generalized linear models regressing scalar outcomes on two‐dimensional image predictors, approximated via the radial B‐spline function. Zhou et al. [17] later extended the generalized linear regression model to incorporate tensor‐structured predictors. They assumed a rank‐R decomposition for the tensor such that the number of parameters to be estimated is substantially reduced. Wang et al. [18] proposed a minimum penalized total variation approach, based on the scalar‐on‐image regression model, that yields piecewise‐smooth regression coefficients. Jiang et al. [19] studied the supervised FPCA to analyze two‐dimensional imaging data and performed risk prediction by incorporating the estimated FPC scores into the Cox model for right‐censored outcomes. Bayesian nonparametric methods have also been developed to estimate scalar‐on‐image regression models; for instance, see Goldsmith et al. [20], Kang et al. [21], and Feng et al. [22]. However, the aforementioned work typically assumes that the image is bounded within a rectangular domain, and the outcome variable is often noncensored or right‐censored. Mattos et al. [23] introduced an estimation approach for the tuning parameter in a semiparametric linear mixed effects model, which incorporates a roughness penalty on the unknown smooth function. A scant amount of work has paid attention to the association between image predictors and interval‐censored outcome variables.

The rest of the paper is structured as follows. Section 2 first introduces the notations, the models for image predictor and survival outcome, and the likelihood. A flexible class of semiparametric transformation models incorporating imaging predictors is proposed for the survival outcomes. A sieve maximum likelihood approach for interval‐censored data with group penalties is proposed for identifying the regions of interest. In Section 3, we discuss the computation algorithms of the penalized estimator. Extensive simulation studies are conducted in Section 4, investigating the finite‐sample performance of the proposed methods. We illustrate the methods based on the ADNI dataset in Section 5. Some concluding remarks are provided in Section 6.

## 2 Model and Likelihood

Let X(s) be a random two‐dimensional stochastic process modeling the spatially correlated pixels of an image, where location $s\equiv \left(s_{1},s_{2}\right)\in \Omega \subset {ℛ}^{2}$, and Ω is a bounded two‐dimensional domain of possibly irregular shape. Without loss of generality, we assume that X(.) has mean 0. Suppose that there are n realizations of the random process, where each realization pertains to a two‐dimensional surface. Furthermore, we observe $N_{i}$ sets of noisy samples from the ith surface, i=1,…,n. The observed data point $Y_{\mathrm{ij}}$ is given by

$$Y_{\mathrm{ij}}=X_{i}\left(s_{\mathrm{ij}}\right)+\epsilon_{\mathrm{ij}},$$

for i=1,…,n and $j=1,\ldots ,N_{i}$ where $E\left(\epsilon_{\mathrm{ij}}\right)=0$, $E\left(\epsilon_{\mathrm{ij}},\epsilon_{ij^{\prime }}\right)=I\left(j=j^{\prime }\right)\sigma^{2}$, $\epsilon_{\mathrm{ij}}$ is independent of $s_{\mathrm{ij}}$, and I(.) is the indicator function. Note that $X_{i}\left(.\right)$ is intrinsically an infinite‐dimensional function measured on a two‐dimensional space with an irregular boundary, which refers to a circular region along the axial plane in the study of neuroimaging data. We approximate $X_{i}\left(.\right)$ via the bivariate polynomial splines constructed on the partitions over a two‐dimensional triangulated domain [13, 14]. Compared with other polygon shapes, triangulation offers the advantage that any polygonal domain of arbitrary shape can be partitioned into finitely many triangles, thus providing greater flexibility. Let $\Delta =\left\{\tau_{1},\ldots ,\tau_{M}\right\}$ be the collection of triangles that forms a triangulation of $\Omega =\cup_{l=1}^{M}\;\tau_{l}$, where each $\tau_{l}$ is the convex hull of three points that are not colinear, and any nonempty intersection between $\tau_{l}$ and $\tau_{l^{\prime }}$ ($l\neq l^{\prime }$) is either a common vertex or a common edge. The spline space of a degree d and smoothness r over triangulation Δ can be defined as ${\left.T_{d}^{r}\left(\Delta \right)=\{u\in C^{r}\left(\Omega \right):u\right|}_{\tau }\in {\mathbb{P}}_{d},\tau \in \Delta \}$ where r≥0, $C^{r}\left(\Omega \right)$ denote the collection of all r‐th continuously differentiable functions over Ω, and ${\mathbb{P}}_{d}$ denotes the space of all polynomials with degree not greater than d.

Specifically, we adopt the Bernstein polynomials, with each polynomial tailored to a specific triangle in Ω. Suppose that τ∈Δ is a nondegenerate triangle (with a nonzero area) with vertices $v_{1},v_{2}$, and $v_{3}$. Then, an arbitrary point s∈Ω can be uniquely represented by $s=b_{1}v_{1}+b_{2}v_{2}+b_{3}v_{3}$, where $\left(b_{1},b_{2},b_{3}\right)$ are known as the barycentric coordinates of the point s relative to triangle τ, and $b_{1}+b_{2}+b_{3}=1$. We define a set of Bernstein polynomial basis functions of degree d for s relative to τ by $B^{\tau ,d}{\left(s\right)}^{T}=\left\{B_{\mathrm{ijk}}^{\tau ,d}\left(s\right),i+j+k=d\right\}$, with dimension $K_{d}=\left(\begin{array}{c} d+2\\ 2 \end{array}\right)$, where $B_{\mathrm{ijk}}^{\tau ,d}\left(s\right)=d!{\left(i!j!k!\right)}^{-1}b_{1}^{i}b_{2}^{j}b_{3}^{k}$. Therefore, the polynomial piece of spline u restricted to τ is given by ${\;u|}_{\tau }=\alpha^{\tau T}B^{\tau ,d}$, where $\alpha^{\tau T}=\left\{\alpha_{\mathrm{ijk}}^{\tau },i+j+k=d\right\}$ is a $K_{d}$‐dimensional vector of coefficients of the basis functions. In the sequel, we assume that d is common to all triangles, and we drop the subscript d in K and superscript d in B for notational simplicity. The Bernstein polynomial based approximation of $X_{i}\left(s\right)$ is given by

(1) $${\tilde{X}}_{i}\left(s\right)=\sum_{l=1}^{M}\;{\alpha_{i}^{\tau_{l}}}^{T}B^{\tau_{l}}\left(s\right).$$

The estimates of $\alpha_{i}={\left({\alpha_{i}^{\tau_{1}}}^{T},\ldots ,{\alpha_{i}^{\tau_{M}}}^{T}\right)}^{T}$ can be obtained by minimizing the least squares criterion with the thin‐plate spline penalty [24, 25]:

(2) $$\sum_{i=1}^{n}\sum_{j=1}^{N_{i}}{\left\{Y_{\mathrm{ij}}-{\tilde{X}}_{i}\left(s_{\mathrm{ij}}\right)\right\}}^{2}+\varsigma_{i}\sum_{\tau \in \Delta }\;\int_{\tau }\;\sum_{i^{\prime }+j^{\prime }=2}\left(\begin{array}{c} 2\\ i^{\prime } \end{array}\right)\;{\left(\nabla_{s_{1}}^{i^{\prime }}\nabla_{s_{2}}^{j^{\prime }}{\tilde{X}}_{i}\right)}^{2}ds_{1}ds_{2},$$

where $\varsigma_{i}>0$ pertains to the roughness penalty parameter, and $\nabla_{s_{1}}^{p}$ pertains to the pth‐order derivative in the direction $s_{1}$ at the point $s=\left(s_{1},s_{2}\right)$. In addition, we require Hα=0 to meet the smoothness condition of the splines, where H is a matrix that enforces smoothness across shared edges of triangles; some examples on the construction of H can be found in Zhou and Pan [26].

In addition to the image data, let T be the failure time, and Z be a vector of demographic covariates. We propose a flexible class of semiparametric transformation models for T with the conditional cumulative hazard function

(3) $$\Lambda \left(t|X_{i},Z_{i}\right)=G\left[\Lambda \left(t\right)\exp\left\{\beta^{T}Z_{i}+\int_{s\in \Omega }\gamma \left(s\right)X_{i}\left(s\right)ds\right\};\rho \right],$$

where G is a prespecified strictly increasing transformation function indexed by parameter ρ, Λ is an unspecified increasing function with Λ(0)=0, β is a vector of regression parameters, and γ(s) is an imaging parameter which characterizes the effects of imaging data on the hazard function. The transformation model in Equation (3) reduces to the Cox proportional hazards (PH) model when G(x;ρ)=x, and the proportional odds (PO) model when G(x;ρ)=log(1+x), respectively. Hence, it is reminiscent of the logarithmic or the Box‐Cox transformation functions. In this paper, we assume the logarithmic transformation function G(x;ρ)=log(1+ρx)/ρ with ρ≥0, where ρ=0 and ρ=1 correspond to the Cox PH and PO models, respectively. Equation (3) equipped with the logarithmic transformation function is also known as the generalized odds rate model [27] in the literature. For mathematical tractability, we approximate $X_{i}\left(s\right)$ in Equation (3) by ${\tilde{X}}_{i}\left(s\right)$ in Equation (1), and suppose that γ(s) can also be expanded on the set of bivariate Bernstein polynomial basis functions, that is $\gamma \left(s\right)=\sum_{l=1}^{M}\;\gamma^{\tau_{l}T}B^{\tau_{l}}\left(s\right)$, where $\gamma^{\tau_{l}}$ is a K‐dimensional vector of coefficients of the basis functions restricted to the lth triangle, l=1,…,M. Then, we can rewrite Equation (3) as

(4) $$\begin{array}{ll} \Lambda \left(t|{\tilde{X}}_{i},Z_{i}\right) & =G\left[\Lambda \left(t\right)\exp\left\{\beta^{T}Z_{i}+\int_{s\in \Omega }\left(\sum_{l=1}^{M}\;\gamma^{\tau_{l}T}B^{\tau_{l}}\left(s\right)\right)\right.\right.\\ & \;\left.\left.\left(\sum_{l^{\prime }=1}^{M}\;{\alpha_{i}^{\tau_{l^{\prime }}}}^{T}B^{\tau_{l^{\prime }}}\left(s\right)\right)ds\right\};\rho \right]\\ & \;=G\left[\Lambda \left(t\right)\exp\left\{\beta^{T}Z_{i}+\sum_{l=1}^{M}\int_{s\in \Omega }\;\gamma^{\tau_{l}T}B^{\tau_{l}}\left(s\right){\alpha_{i}^{\tau_{l}}}^{T}B^{\tau_{l}}\left(s\right)ds\right\};\rho \right]\\ & \;=G\left[\Lambda \left(t\right)\exp\left\{\beta^{T}Z_{i}+\sum_{l=1}^{M}\;\gamma^{\tau_{l}T}W^{\tau_{l}}\alpha_{i}^{\tau_{l}}\right\};\rho \right], \end{array}$$

where $W^{\tau_{l}}=\left\langle B^{\tau_{l}},B^{\tau_{l}T}\right\rangle$ is a K×K dimensional matrix, and ⟨⋅,⋅⟩ denotes the inner product of functions. The second last line of Equation (4) is obtained based on the fact that $\left\langle B^{\tau_{l}},B^{\tau_{l^{\prime }}T}\right\rangle =0$ for any $l^{\prime }\neq l$. Let $W=\mathrm{diag}\left(W^{\tau_{1}},\ldots ,W^{\tau_{M}}\right)$ be a block diagonal and positive definite matrix, and $\xi_{i}={\mathrm{W\alpha}}_{i}$. Then, Equation (4) becomes

(5) $$\Lambda \left(t|{\tilde{X}}_{i},Z_{i}\right)=G\left\{\Lambda \left(t\right)e^{\beta^{T}Z_{i}+\gamma^{T}\xi_{i}};\rho \right\},$$

where $\gamma ={\left(\gamma^{\tau_{1}T},\ldots ,\gamma^{\tau_{M}T}\right)}^{T}$.

Suppose $T_{i}$ is censored by a random interval $\left(L_{i},R_{i}\right)$, i=1,…,n. In the framework, we set $L_{i}=0$ and $R_{i}=\infty$ to accommodate left‐ and right‐censored observations. Under the paradigm of noninformative censoring, and for given $\xi_{i}$'s, the observed likelihood of (Λ,β,γ) is proportional to

$$\prod_{i=1}^{n}\left[e^{-G\left\{\Lambda \left(L_{i}\right)e^{\beta^{T}Z_{i}+\gamma^{T}\xi_{i}};\rho \right\}}-e^{-G\left\{\Lambda \left(R_{i}\right)e^{\beta^{T}Z_{i}+\gamma^{T}\xi_{i}};\rho \right\}}\right].$$

Since Λ is modeled nonparametrically, we propose a sieve maximum likelihood approach for parameter estimation. We approximate Λ(t) via $\tilde{\Lambda }\left(t\right)=\sum_{j=1}^{J}\omega_{j}I_{j}\left(t\right)$, where $I_{1},\ldots ,I_{J}$ are the I‐spline basis functions [28] constructed over $\left[0,t_{\max}\right]$, and $\omega =\left(\omega_{1},\ldots ,\omega_{J}\right)$ are nonnegative coefficients ensuring that $\tilde{\Lambda }$ is a monotone function. In practice, $t_{\max}$ is set to be the largest finite value of the combined vector of $L_{i}$'s and $R_{i}$'s. The observed likelihood of θ≡(ω,β,γ) under sieve approximation is given by

(6) $$L\left(\theta \right)=\prod_{i=1}^{n}\left[e^{-G\left\{\sum_{j=1}^{J}\omega_{j}I_{j}\left(L_{i}\right)e^{\beta^{T}Z_{i}+\gamma^{T}\xi_{i}};\rho \right\}}-e^{-G\left\{\sum_{j=1}^{J}\omega_{j}I_{j}\left(R_{i}\right)e^{\beta^{T}Z_{i}+\gamma^{T}\xi_{i}};\rho \right\}}\right].$$

In general, not all regions of the image are of clinical interest, and we wish to identify some useful regions for disease prognosis. We adopt the group lasso [29, 30] to penalize the parameters associated with each of the M triangles. Specifically, we propose to maximize the following objective function:

(7) $$\log L\left(\theta \right)-\lambda \sum_{l=1}^{M}\;s\left(K_{l}\right)∥\gamma^{\tau_{l}}{∥}_{2},$$

where $∥.{∥}_{2}$ denotes the Euclidean norm, $K_{1}=\cdots =K_{M}=K$, s(.) is a function used to rescale the penalty corresponding to the dimensionality of $\gamma^{\tau_{l}}$, and λ≥0 is a tuning parameter that controls the size of the penalty. As is customary, we set $s\left(K_{l}\right)=\sqrt{K}$ pertaining to a normalizing constant for l=1,…,M, and we normalize each element in $\xi_{i}$ before carrying out the estimation procedure. The demographic variables $Z_{i}$ are not penalized in this setting. The group lasso penalty acts as an intermediate between the $l_{1}$‐ and $l_{2}$‐type penalties, selecting variables at the group level. Consequently, if the lth triangle is not penalized, then all values in $\gamma^{\tau_{l}}$ remain nonzero, retaining all the basis functions restricted to that triangle.

## 3 Computational Details

We propose a two‐step estimation procedure for computational efficiency. The estimation in Equation (2) can be handled easily via some existing software, such as the BPST package in R. Given the estimates of $\xi_{i}$'s, we then approximate θ by maximizing the penalized likelihood in Equation (7) with the detailed procedures as follows.

To simplify the computation, we adopt a class of frailty‐induced transformation models [31]. Let $G\left(x;\rho \right)=-\log\Phi_{\zeta }\left(x;\rho \right)$ where $\Phi_{\zeta }\left(x;\rho \right)=\int_{0}^{\infty }\exp\left(-\zeta x\right)f_{\zeta }\left(\zeta ;\rho \right)d\zeta$ is the Laplace transform of a positive‐valued frailty ζ, and $f_{\zeta }\left(.;\rho \right)$ is the density function of ζ. We obtain the logarithmic transformation G(x;ρ)=log(1+ρx)/ρ when ζ follows a gamma distribution with mean 1 and variance ρ. Furthermore, let $\Delta_{\mathrm{Li}}$, $\Delta_{\mathrm{Ri}}$, and $\Delta_{\mathrm{Ii}}\equiv 1-\Delta_{\mathrm{Li}}-\Delta_{\mathrm{Ri}}$ be the indicators for left‐, right‐, and interval‐censored observations, respectively. Then, Equation (6) can be written as

(8) $$\begin{array}{ll} & \prod_{i=1}^{n}\int {\left\{1-e^{-\sum_{j=1}^{J}\zeta_{i}\omega_{j}I_{j}\left(R_{i}\right)e^{\beta^{T}Z_{i}+\gamma^{T}\xi_{i}}}\right\}}^{\Delta_{\mathrm{Li}}}\\ & \;{\left\{e^{-\sum_{j=1}^{J}\zeta_{i}\omega_{j}I_{j}\left(L_{i}\right)e^{\beta^{T}Z_{i}+\gamma^{T}\xi_{i}}}-e^{-\sum_{j=1}^{J}\zeta_{i}\omega_{j}I_{j}\left(R_{i}\right)e^{\beta^{T}Z_{i}+\gamma^{T}\xi_{i}}}\right\}}^{\Delta_{\mathrm{Ii}}}\\ & \;\times {\left\{e^{-\sum_{j=1}^{J}\zeta_{i}\omega_{j}I_{j}\left(L_{i}\right)e^{\beta^{T}Z_{i}+\gamma^{T}\xi_{i}}}\right\}}^{\Delta_{\mathrm{Ri}}}f_{\zeta }\left(\zeta_{i};\rho \right)d\zeta_{i}, \end{array}$$

where $f_{\zeta }\left(.;\rho \right)$ is the density of a gamma distribution with both shape and rate parameters equal to $\rho^{-1}$. The maximization of Equation (8) is challenging due to the lack of analytical expressions for $\omega_{1},\ldots ,\omega_{J}$. To address this issue and accommodate the latent variable $\zeta_{i}$, we propose a data augmentation approach and develop an EM algorithm for obtaining the sieve maximum likelihood estimate (MLE). For i=1,…,n and j=1,…,J, let $A_{\mathrm{ij}}$ and $B_{\mathrm{ij}}$ be two latent Poisson variables that are independent given $\zeta_{i}$, with conditional means $\zeta_{i}\mu_{\mathrm{ij}}$ and $\zeta_{i}\eta_{\mathrm{ij}}$ respectively, where $\mu_{\mathrm{ij}}\equiv \omega_{j}\left\{\Delta_{\mathrm{Li}}I_{j}\left(R_{i}\right)+\Delta_{\mathrm{Ii}}I_{j}\left(L_{i}\right)\right\}e^{\beta^{T}Z_{i}+\gamma^{T}\xi_{i}}$ and $\eta_{\mathrm{ij}}\equiv \omega_{j}\left[\Delta_{\mathrm{Ii}}\left\{I_{j}\left(R_{i}\right)-I_{j}\left(L_{i}\right)\right\}+\Delta_{\mathrm{Ri}}I_{j}\left(L_{i}\right)\right]e^{\beta^{T}Z_{i}+\gamma^{T}\xi_{i}}$. Furthermore, let $A_{i}\equiv \sum_{j=1}^{J}A_{\mathrm{ij}}$, $B_{i}\equiv \sum_{j=1}^{J}B_{\mathrm{ij}}$, and

$${\tilde{O}}_{i}^{T}=\left\{\begin{array}{ll} A_{i}>0,B_{i}=0, & \text{if}\;\Delta_{\mathrm{Li}}=1;\\ A_{i}=0,B_{i}>0, & \text{if}\;\Delta_{\mathrm{Ii}}=1;\\ A_{i}=0,B_{i}=0, & \text{otherwise.} \end{array}\right.\;$$

One can see that the observed likelihood in Equation (8) is equivalent to that obtained from the data $\left(O_{1},\ldots ,O_{n}\right)$ where $O_{i}\equiv \left(L_{i},R_{i},Z_{i},\xi_{i},{\tilde{O}}_{i}^{T}\right)$, treating $A_{\mathrm{ij}}$, $B_{\mathrm{ij}}$, and $\zeta_{i}$ as missing data. Therefore, the maximizer of Equation (8) can be computed based on the EM algorithm with the augmented data. It suffices to write down the complete data log‐likelihood

$$\ell_{C}\left(\theta \right)=\sum_{i=1}^{n}\sum_{j=1}^{J}\left(-\mu_{\mathrm{ij}}\zeta_{i}+A_{\mathrm{ij}}\log\mu_{\mathrm{ij}}-\eta_{\mathrm{ij}}\zeta_{i}+B_{\mathrm{ij}}\log\eta_{\mathrm{ij}}\right),$$

omitting the terms which do not depend on θ.

Let $\theta^{\left(d\right)}$ denote the current estimates of θ in the dth iteration (d=0,1,…) of the EM algorithm. In the E‐step, we evaluate $\hat{E}\left(A_{\mathrm{ij}}\right)$, $\hat{E}\left(B_{\mathrm{ij}}\right)$, and $\hat{E}\left(\zeta_{i}\right)$ where $\hat{E}\left(\cdot \right)$ denotes the conditional expectation given $\left(O_{1},\ldots ,O_{n}\right)$ evaluated at $\theta^{\left(d\right)}$. The analytical expressions of these conditional expectations are relegated to the Appendix A, where we show that only conditional expectations of functions of $\zeta_{i}$ are required in the E‐step. In particular, those integrals have closed‐form expressions, thus, the computationally intensive numerical approximation is not required in the proposed method.

In the M‐step, we obtain $\theta^{\left(d+1\right)}$ by maximizing the penalized Q‐function $Q\left(\theta ,\theta^{\left(d\right)}\right)\equiv \hat{E}\left(\ell_{C}\left(\theta \right)\right)-\lambda \sum_{l=1}^{M}\;s\left(K_{l}\right)∥\gamma^{\tau_{l}}{∥}_{2}$. Taking the partial derivative of $Q\left(\theta ,\theta^{\left(d\right)}\right)$ with respect to ω and setting it to zero yields a closed‐form solution for ω, in terms of β and γ, as

$$\begin{array}{ll} \omega_{j}^{*}\left(\beta ,\gamma \right) & =\frac{\sum_{i=1}^{n}\Delta_{\mathrm{Li}}\hat{E}\left(A_{\mathrm{ij}}\right)+\Delta_{\mathrm{Ii}}\hat{E}\left(B_{\mathrm{ij}}\right)}{\sum_{i=1}^{n}e^{\beta^{T}Z_{i}+\gamma^{T}\xi_{i}}\hat{E}\left(\zeta_{i}\right)\left\{\Delta_{\mathrm{Ri}}I_{j}\left(L_{i}\right)+\left(1-\Delta_{\mathrm{Ri}}\right)I_{j}\left(R_{i}\right)\right\}},\\ & \;\text{for}\;j=1,\ldots ,J. \end{array}$$

By replacing $\omega_{j}$ with $\omega_{j}^{*}\left(\beta ,\gamma \right)$ in $Q\left(\theta ,\theta^{\left(d\right)}\right)$, the revised penalized Q‐function is

(9) $$\hat{E}\left(\ell_{C}\left(\omega^{*},\beta ,\gamma \right)\right)-\lambda \sum_{l=1}^{M}s\left(K_{l}\right)∥\gamma^{\tau_{l}}{∥}_{2},$$

where $\hat{E}\left(\ell_{C}\left(\omega^{*},\beta ,\gamma \right)\right)$ is given by

$$\begin{array}{ll} & \sum_{i=1}^{n}\sum_{j=1}^{J}\left(\beta^{T}Z_{i}+\gamma^{T}\xi_{i}-\log\left[\sum_{k=1}^{n}e^{\beta^{T}Z_{k}+\gamma^{T}\xi_{k}}\hat{E}\left(\zeta_{k}\right)\left.\Delta_{\mathrm{Rk}}I_{j}\left(L_{k}\right)\right\}\right.\right.\\ & \left.\left.\left.+\left(1-\Delta_{\mathrm{Rk}}\right)I_{j}\left(R_{k}\right)\right\}\right]\right)\left\{\Delta_{\mathrm{Li}}\hat{E}\left(A_{\mathrm{ij}}\right)+\Delta_{\mathrm{Ii}}\hat{E}\left(B_{\mathrm{ij}}\right)\right\}. \end{array}$$

Based on Equation (9), $\beta^{\left(d+1\right)}$ is obtained via a one‐step Newton–Raphson algorithm. Then, we update γ via the block coordinate descent algorithm [30, 32]. Specifically, we fix the values of $\gamma^{\tau_{l^{\prime }}}$ for any $l^{\prime }\neq l$, and (ω,β) at $\left(\omega^{\left(d\right)},\beta^{\left(d+1\right)}\right)$, while updating $\gamma^{\tau_{l}}$. Based on the direct consequence of the Karush–Kuhn–Tucker conditions, we have $\gamma^{\tau_{l}^{\left(d+1\right)}}=0$ if $∥G_{l}{∥}_{2}\leq \lambda s\left(K_{l}\right)$ where ${\left.G_{l}=\left\{\frac{\partial }{\partial \gamma^{\tau_{l}}}\hat{E}\left(\ell_{C}\left(\omega^{*},\beta ,\gamma \right)\right)\right\}\right|}_{\gamma^{\tau_{l}}=0}$, and $\gamma^{\tau_{l}^{\left(d+1\right)}}$ is obtained via minimizing the negative penalized‐Q function in Equation (9) otherwise, for l=1,…,M. Then, we obtain $\omega_{j}^{\left(d+1\right)}=\omega_{j}^{*}\left(\beta^{\left(d+1\right)},\gamma^{\left(d+1\right)}\right)$ for j=1,…,J. We iterate between the E‐ and M‐steps until convergence, where the maximum absolute difference between two consecutive estimates is smaller than a certain threshold.

Determination of the tuning parameter λ is a crucial task in the estimation procedure. We propose to implement a data‐adaptive Akaike information criterion (AIC):

(10) $${\mathrm{AIC}}_{\lambda }=-2\log L\left({\hat{\theta }}_{\lambda }\right)+2{\mathrm{df}}_{\lambda },$$

where ${\hat{\theta }}_{\lambda }$ is the estimator obtained in Equation (7) under a prespecified λ, and the degree of freedom ${\mathrm{df}}_{\lambda }$ is taken as the total number of nonzero estimates in ${\hat{\theta }}_{\lambda }$. In practice, we consider a grid of values for λ and choose the model with a minimum value of ${\mathrm{AIC}}_{\lambda }$. This criterion performs well as indicated in our simulation studies. Other model selection methods, such as the Bayesian information criterion [33] or cross‐validation, can also be considered.

## 4 Simulation Study

**FIGURE 1:**
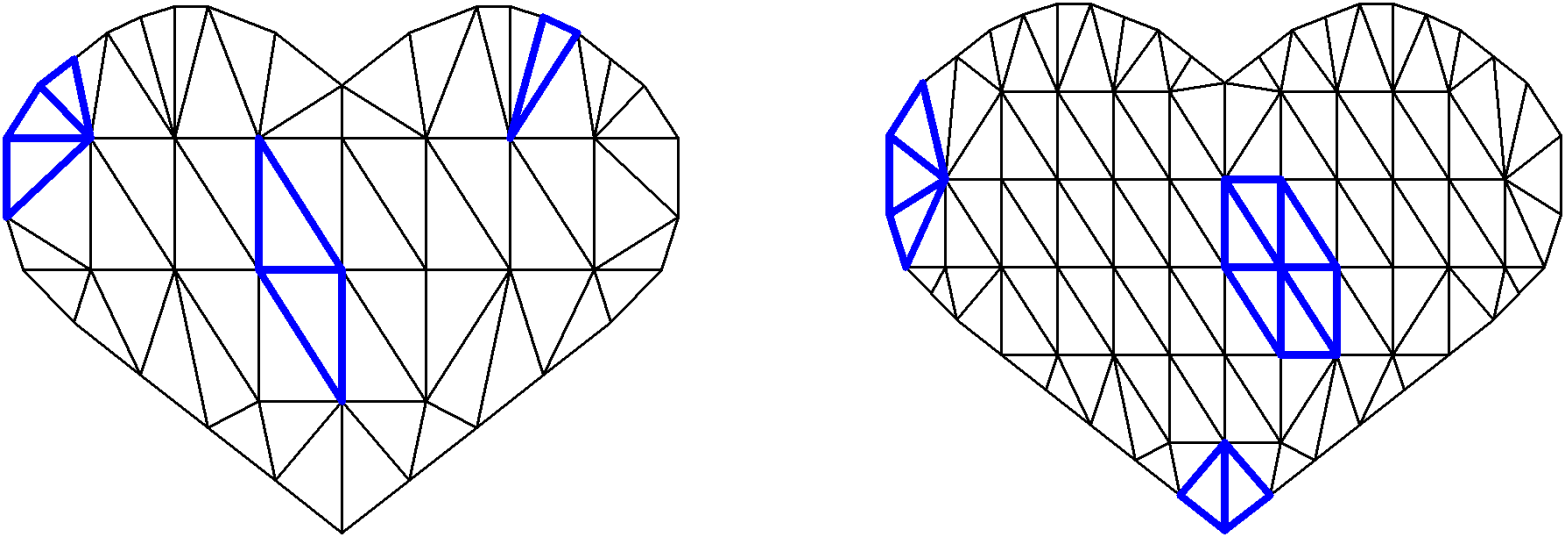
Illustration of the triangulation grids used in the simulation study. The left and right panels displayed grids of 62 and 118 triangles, respectively. The triangles with thickened boundaries illustrate the mechanism by which a certain proportion of the triangles is selected to be associated with the survival outcome.

We study the finite‐sample performance of the proposed methods to identify the regions of interest. To mimic the practical situation in which images are observed with an irregular boundary, we confine the images to a rectangular grid of size 40 × 40, where 1036 of them are situated within a heart‐shaped boundary region. We construct the sets of bivariate Bernstein polynomials based on two settings on the fineness of the triangulation, namely M=62 triangles with 47 vertices, and M=118 triangles with 80 vertices. The triangulation grids used in these two settings are depicted in Figure 1. Specifically, the jth pixel of the image surface observed for the ith subject is given by

$$Y_{\mathrm{ij}}=\sum_{l=1}^{M}\;{\alpha_{i}^{\tau_{l}}}^{T}B^{\tau_{l}}\left(s_{\mathrm{ij}}\right)+\epsilon_{\mathrm{ij}},$$

for i=1,…,n; $j=1,\ldots ,N_{i}$, and $N_{i}=1036$ for all i. Specifically, we set the degree of the Bernstein polynomial d=2 (i.e., K=6) and $\epsilon_{\mathrm{ij}}$'s are generated from a normal distribution with mean 0 and variance $0.1^{2}$. We generate $\alpha_{i}$ from a zero‐mean multivariate normal distribution with covariance matrix ∑ where the diagonal elements of ∑ are equal to 1, and the within‐triangle and between‐triangle correlations are equal to 0.4 and 0.1, respectively. Subsequently, we generate the survival time $T_{i}$ based on the model:

$$\Lambda \left(t|W,\alpha_{i},Z_{i}\right)=G\left[\Lambda \left(t\right)\exp\left\{\beta^{T}Z_{i}+\sum_{l\in S_{l}}\;\gamma^{\tau_{l}T}W^{\tau_{l}}\alpha_{i}^{\tau_{l}}\right\};\rho \right],$$

where $S_{l}$ is a set of indices of the triangles that are assigned to be associated with the survival outcomes. We set the basis coefficient $\gamma^{\tau_{l}}=\left(0.1,0.2,0.3,0.5,0.6,0.4\right)$ for all $l\in S_{l}$, and $\gamma^{\tau_{l}}=0$ otherwise. We set $\Lambda \left(t\right)=t^{2}/4$, and the transformation parameter ρ to be 0,0.5 and 1. The interarrival times follow a uniform distribution with support (0.1,0.5). We assume that there is an administrative censoring time, such that observations are right‐censored beyond this time point. The average right‐censoring proportion varies from 30% to 45% across the scenarios. We set β=(0.5,−0.5) and $Z_{i}=\left(Z_{i1},Z_{i2}\right)$ where $Z_{i1}$ follows a Bernoulli distribution with probability 0.5 and $Z_{i2}$ follows the standard normal distribution. We consider n=100,200,500, and 1000, and the number of selected triangles $|S_{l}|$ to be ⌊aM⌋, the largest integer value smaller than or equal to aM, where a=0.025,0.05, and 0.1.

We adopt the I‐splines basis functions with degree 3 and 4 interior knots (i.e., J=7) to approximate Λ. The knots were positioned at the empirical quantiles of the endpoints of the observed intervals. For implementing the EM algorithm, we set the initial parameter values as follows: $\omega_{j}^{\left(0\right)}=0.1$ for j=1,…,J, $\beta^{\left(0\right)}=0$, and $\gamma^{\left(0\right)}=0$. The tolerance level for the convergence criterion is set to be $10^{-3}$. The optimal value of λ is selected by AIC, as discussed in Section 3.

Table 1 presents the simulation results for two different triangulation grid settings, with 100 replicates in each scenario. The performance of the proposed methods is assessed based on the true positive rate and false positive rate. The true positive rate is defined as the proportion of triangles in $S_{l}$ being correctly identified, while the false positive rate is the proportion of triangles which does not belong to $S_{l}$ but are identified. The two rates are computed for each replicate, and the average rates are reported. The empirical results demonstrate that the proposed method performs satisfactorily under the transformation models in general, except when the sample size is as small as n=100 and when the number of triangles in $S_{l}$ is relatively large, such as when a=0.1, as expected. This indicates that the proposed method can effectively identify all the regions of interest that are truly associated with survival time. Also, the false positive rates remain consistently low across all scenarios, suggesting that the proposed method often accurately excludes regions without any impact during the variable selection process.

**TABLE 1:** Empirical true positive (TP) and false positive (FP) for identifying regions of interest with different numbers of triangulation grids M, the proportion of selected triangles a, and sample sizes n for ρ=0 (PH model), ρ=0.5, and ρ=1 (PO model).

		M=62						M=118					
a=0.025		a=0.05		a=0.1		a=0.025		a=0.05		a=0.1	
ρ	n	TP	FP	TP	FP	TP	FP	TP	FP	TP	FP	TP	FP
0	100	1.000	0.003	0.847	0.007	0.597	0.022	0.995	0.002	0.816	0.014	0.143	0.003
	200	1.000	0.004	1.000	0.012	0.963	0.040	1.000	0.002	0.998	0.016	0.645	0.032
	500	1.000	0.010	1.000	0.009	1.000	0.030	1.000	0.002	1.000	0.013	0.874	0.039
	1000	1.000	0.029	1.000	0.015	1.000	0.035	1.000	0.008	1.000	0.014	0.929	0.040
0.5	100	0.990	0.003	0.850	0.008	0.578	0.018	0.995	0.003	0.730	0.012	0.122	0.004
	200	1.000	0.004	1.000	0.013	0.970	0.037	1.000	0.002	1.000	0.019	0.650	0.030
	500	1.000	0.007	1.000	0.006	1.000	0.028	1.000	0.003	1.000	0.012	0.894	0.044
	1000	1.000	0.019	1.000	0.014	1.000	0.031	1.000	0.004	1.000	0.013	0.940	0.050
1.0	100	0.980	0.002	0.877	0.009	0.575	0.019	0.980	0.002	0.798	0.012	0.097	0.003
	200	1.000	0.003	0.997	0.010	0.960	0.041	1.000	0.002	0.994	0.015	0.564	0.020
	500	1.000	0.006	1.000	0.009	0.998	0.030	1.000	0.003	1.000	0.011	0.878	0.041
	1000	1.000	0.012	1.000	0.008	1.000	0.031	1.000	0.003	1.000	0.009	0.935	0.049

**FIGURE 2:**
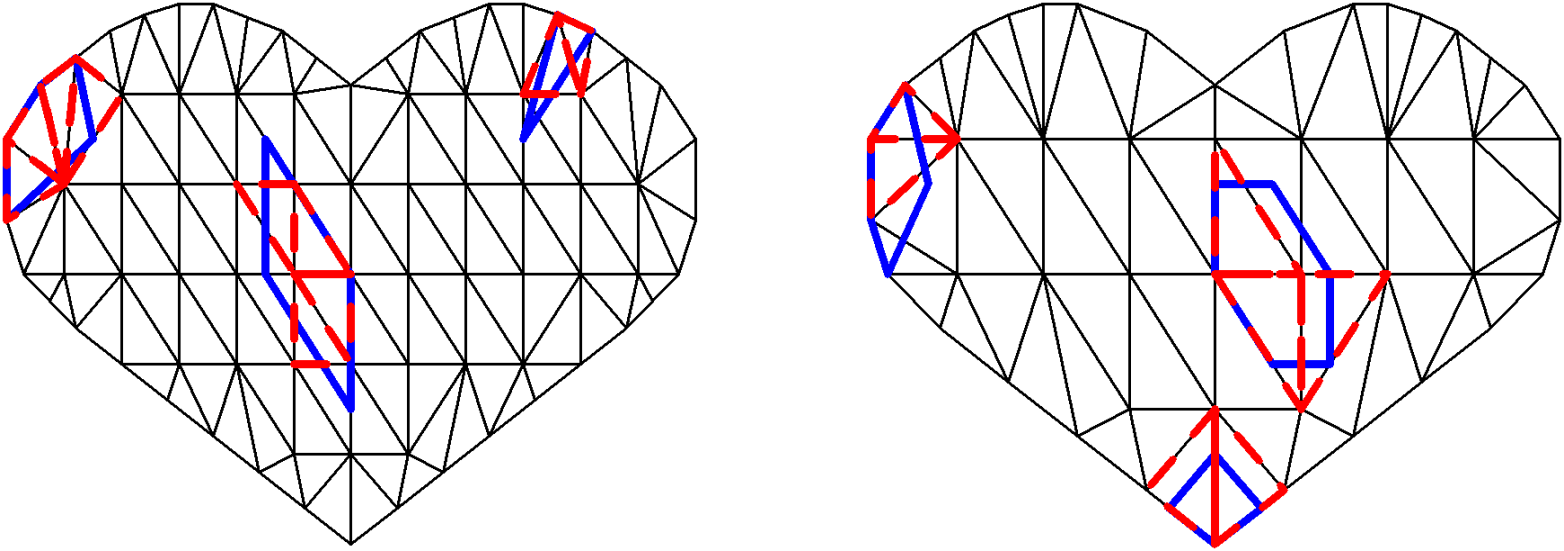
Illustration of the surrogate triangles to facilitate the definition of true and false positive rates under a misspecified triangulation grid in the simulation. In the left (right) panel, the blue solid lines indicate the triangles associated with the survival outcome with triangulation grids M=62 (M=118), and the red dashed lines indicate the surrogate triangles associated with the survival outcome under a misspecified grid where $M^{*}=118$ ($M^{*}=62$).

To assess the potential impact of an incorrectly specified triangulation grid on the performance of the proposed methods, we conduct a simulation where the grid used for data generation differs from the grid used for identifying regions of interest. Specifically, data are generated using a (true) grid with M=62 triangles, with those associated with survival outcomes depicted in the left panel of Figure 1. We then fit the model using a (misspecified) triangulation grid with $M^{*}=118$ triangles, and vice versa. All other parameter settings remain unchanged as above. As our main objective is to evaluate the precision of the identification methods, it necessitates a surrogate measurement for determining true and false positive rates under the conditions of misspecified grids. To achieve this, we examine each triangle within the misspecified grid and classify it as associated with the outcome if its centroid falls within the region defined by the true grid. Note that varying triangle proportions a in the true grid yield different numbers of (associated) surrogate triangles in the misspecified grid, leading to different surrogate proportions $a^{*}$. Figure 2 illustrates the surrogate triangles, marked with red dashed lines, which are utilized to assess the surrogate performance of the methods, alongside the true region of interest bounded with blue solid lines. The results, along with the values of M, $M^{*}$, a, and $a^{*}$ in the settings, are summarized in Table 2.

**TABLE 2:** Empirical true positive (TP) and false positive (FP) for identifying regions of interest under misspecified triangulation grids, the proportion of selected triangles a, and sample sizes n for ρ=0 (PH model), ρ=0.5, and ρ=1 (PO model). Here, “prop” stands for proportion.

True grid		M=62						M=118					
True prop		a=0.025		a=0.05		a=0.1		a=0.025		a=0.05		a=0.1	
Adopted grid		$M^{*}=118$						$M^{*}=62$					
Surrogate prop		$a^{*}=0.017$		$a^{*}=0.051$		$a^{*}=0.085$		$a^{*}=0.032$		$a^{*}=0.065$		$a^{*}=0.113$	
ρ	n	TP	FP	TP	FP	TP	FP	TP	FP	TP	FP	TP	FP
0	100	0.470	0.006	0.370	0.007	0.241	0.006	0.985	0.008	0.772	0.034	0.324	0.021
	200	0.505	0.009	0.677	0.016	0.590	0.024	1.000	0.007	0.973	0.041	0.713	0.057
	500	0.505	0.010	0.808	0.022	0.770	0.048	1.000	0.020	0.995	0.042	0.856	0.083
	1000	0.500	0.012	0.830	0.023	0.819	0.050	1.000	0.051	1.000	0.050	0.899	0.096
0.5	100	0.375	0.005	0.370	0.006	0.187	0.006	0.985	0.005	0.807	0.031	0.303	0.021
	200	0.550	0.009	0.708	0.018	0.599	0.026	1.000	0.006	0.948	0.036	0.659	0.058
	500	0.500	0.010	0.830	0.023	0.782	0.043	1.000	0.015	1.000	0.039	0.846	0.084
	1000	0.500	0.010	0.847	0.022	0.840	0.055	1.000	0.035	1.000	0.047	0.876	0.095
1.0	100	0.340	0.004	0.308	0.006	0.148	0.004	0.980	0.006	0.760	0.031	0.244	0.017
	200	0.530	0.008	0.723	0.018	0.593	0.024	1.000	0.007	0.948	0.036	0.687	0.060
	500	0.515	0.010	0.832	0.022	0.778	0.040	1.000	0.014	0.998	0.034	0.850	0.092
	1000	0.500	0.010	0.847	0.022	0.823	0.051	1.000	0.029	1.000	0.044	0.877	0.097

When an overly refined grid is erroneously applied to the data, specifically when the true grid size is M=62 but a grid size of $M^{*}=118$ is applied, the true positive rates tend to diminish. This decline is likely attributable to the discordance between the true and misspecified grids, as well as a decrease in the sparsity of the associated triangles, given that the number of surrogate triangles exceeds that of the true triangles. Conversely, when a coarser grid is mistakenly applied, where the true grid size is M=118 but a grid size of $M^{*}=62$ is used, the true positive rates remain satisfactory. This result may be due to an increase in the sparsity of the associated triangles. Overall, the false positive rates exhibit a moderate increase, particularly when the adopted grid is coarser than the true grid, compared to scenarios where the triangulation grids are correctly specified. The results demonstrate that the proposed methods still manage to provide reasonable performance, although careful selection of the triangulation grid can enhance precision. In practice, the optimal choice of M is unknown. Adopting a balanced triangulation grid guided by practitioners, that is both clinically meaningful and tailored to the specific context of the study, is recommended to enhance the performance of the identification methods. Some guidelines for the choice of triangulation can be found in Wang et al. [25].

## 5 Application to AD Study

We demonstrate the proposed methods using the ADNI dataset [34]. This is a longitudinal study in which the cognitive status of the subjects was reassessed approximately every 6 months in the first 2 years, followed by annual evaluations beyond 2 years. The maximum number of visit time points was 13, and the maximum follow‐up period was 120 months. At each visit time point, the subjects underwent brain MRI scans and were clinically classified into one of the three stages of AD, namely cognitive normal (CN), mild cognitive impairment (MCI), and AD. We treat the onset of AD as the event of interest, which was interval‐censored due to the study design. The main objective of this analysis is to identify the region of interest in the brain MRI scan associated with the survival time of the patients. We focus on a subset of subjects initially diagnosed as CN or MCI (i.e., AD‐free) at baseline. This yields a sample size of n=674 subjects of which 286 have been diagnosed with AD by the end of the study. Apart from the volumetric images at baseline, other covariates, including gender and age at baseline, are available for each subject. Before the statistical analysis, the MRI scans are preprocessed to ensure that they are approximately transformed into the same stereotaxic space, based on the registration procedure in Ma et al. [35]. We proceed to extract the middle slice from both the axial and sagittal axes of the processed image and store the pixel intensities of the slices in the format of a 200×200 matrix.

**FIGURE 3:**
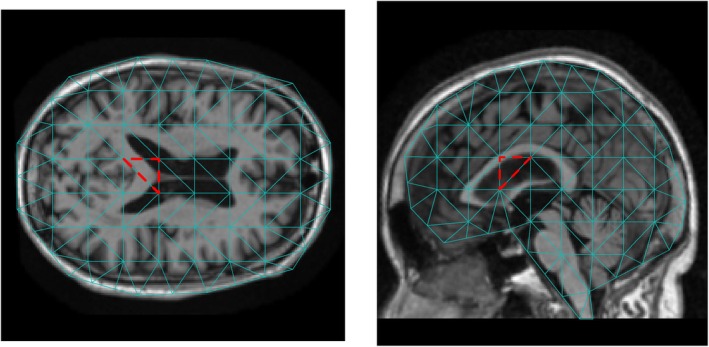
The triangulation over the region of the brain. Left panel: Axial plane with M=118 triangles. Right panel: Sagittal plane with M=103 triangles.

As illustrated in Figure 3, we construct boundaries to envelop the brain regions on the axial and sagittal planes in the MRI scans and partition the regions into M=118 and M=103 triangles, respectively. For i=1,…,674, a total number of $N_{i}=21,393$ data points fall inside the boundary of the axial plane, constituting $Y_{\mathrm{ij}}$'s, whereas there are $N_{i}=16,078$ data points inside the boundary for the sagittal plane. We let gender and age‐at‐baseline be the unpenalized covariates Z. We adopt the I‐splines basis functions with degree 3 and 4 interior knots. We adopt the same set of initial parameter values for the EM algorithm as in the simulation study. The tolerance level for the convergence of the EM algorithm is set to be $10^{-4}$. The degree of the Bernstein polynomial d and the transformation parameter ρ are typically unknown in practice. To achieve a parsimonious fit, we perform a two‐dimensional grid search over the parameters (d,ρ). Specifically, we vary ρ from 0 to 1.5 in increments of 0.1, and we consider d values from the set {1,2,3,4}. For each pair (d,ρ), we compute the minimum value of ${\mathrm{AIC}}_{\lambda }$, where ${\mathrm{AIC}}_{\lambda }$ is defined in Equation (10) based on a grid of λ values ranging from $10^{1}$ to $10^{2.5}$. The interval (1,2.5) is divided into 500 uniformly spaced partitions, serving as exponents with base 10. We then identify the optimal model by selecting the pair (d,ρ) that yields the smallest AIC value among all evaluated combinations. In terms of computational burden, the EM algorithm takes approximately 30 min for computing the 500 ${\mathrm{AIC}}_{\lambda }$ values for d=2 and a given value of ρ in each of the planes using a standard desktop (2.4GHz, 16GB RAM) without parallel programming.

**FIGURE 4:**
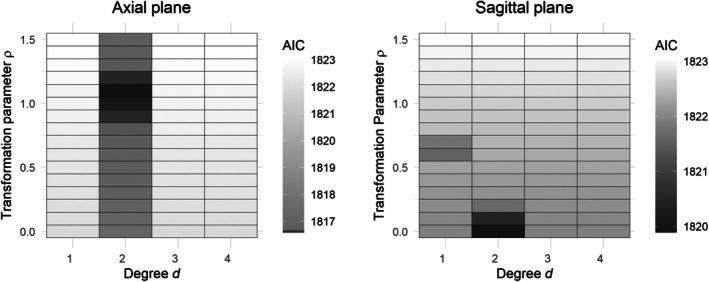
The minimum value of ${\mathrm{AIC}}_{\lambda }$ achieved in each given pair of (d,ρ) for the axial and sagittal planes.

The results are illustrated in Figure 4. One can observe that the model with d=2 and ρ=1.1 achieves the lowest AIC value for the axial plane, while the model with d=2 and ρ=0 achieves the lowest AIC value for the sagittal plane. This suggests that the PO and PH models should be applied to the data for axial and sagittal planes, respectively. The selected model identifies only one triangle associated with survival time in both planes. The selected triangles are marked by red dotted lines in the two panels of Figure 3, and they roughly correspond to the lateral ventricle region of the brain. The change in the size of the ventricle is known to be highly associated with impairment in cognitive function and dementia. Enlargement of the ventricle region has been identified as a reliable predictor for AD progression [4, 36].

To assess the model predictive performance of the proposed method, we use a 10‐fold internal cross‐validation procedure based on the integrated Brier score (IBS). The IBS, introduced by Graf et al. [37], is a prominent and widely adopted tool for gauging prediction error for right‐censored data. Tsouprou et al. [38] later extended this method to accommodate interval‐censored data. Specifically, it takes the form:

$$\mathrm{IBS}=\frac{1}{n}\sum_{i=1}^{n}\frac{1}{t_{\max}}\int_{0}^{t_{\max}}{\left\{I\left(T_{i}>t\right)-{\hat{S}}_{i}(t|{\tilde{X}}_{i},Z_{i})\right\}}^{2}dt,$$

where ${\hat{S}}_{i}\left(t|{\tilde{X}}_{i},Z_{i}\right)$ is the estimated survival function of the ith subject evaluated at time t. Clearly, $I\left(T_{i}>t\right)=1$ for $t\leq L_{i}$ and $I\left(T_{i}>t\right)=0$ for $t>R_{i}$. When $L_{i}<t\leq R_{i}$, $I\left(T_{i}>t\right)$ is unknown and is estimated by

$$\hat{I}\left(T_{i}>t\right)=\frac{{\hat{S}}_{i}\left(t|{\tilde{X}}_{i},Z_{i}\right)-{\hat{S}}_{i}\left(R_{i}|{\tilde{X}}_{i},Z_{i}\right)}{{\hat{S}}_{i}\left(L_{i}|{\tilde{X}}_{i},Z_{i}\right)-{\hat{S}}_{i}\left(R_{i}|{\tilde{X}}_{i},Z_{i}\right)}.$$

**FIGURE 5:**
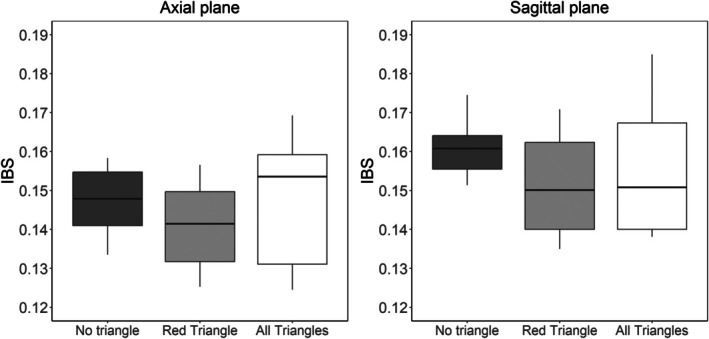
The box plot for the IBS obtained in the 10‐fold internal cross‐validation based on the models (i) without any triangles; (ii) with the selected red triangle; and (iii) all triangles, over the axial and sagittal planes.

It is remarked that IBS ranges between 0 and 1, and a smaller value of this metric reflects a higher model predictive accuracy. In all models used for comparison, we always include the unpenalized covariates $Z_{i}$. For the axial plane, we fit the following three PO models (i) without any triangles, setting γ=0 in Equation (5); (ii) with the selected triangle highlighted in red in Figure 3, based on a second‐stage model, by allowing only $\gamma^{\tau_{l}}$ to be nonzero for that specific triangle in Equation (4); and (iii) with all triangles, permitting all elements in γ to be nonzero in Equation (5). We perform an analogous model comparison for the sagittal plane based on the PH model. The results of the cross‐validation are summarized by box plots in Figure 5. For the axial plane, the average IBS values for models (i), (ii), and (iii) are 0.147, 0.141, and 0.148, respectively. For the sagittal plane, the average IBS values for the three models are 0.161, 0.152, and 0.156, respectively. These results underscore the superior predictive performance of the proposed model with the selected triangle in red over the two planes, as evidenced by its minimal average IBS.

## 6 Conclusions and Discussion

Brain MRI is pivotal in prognostic assessments for AD, yet integrating it into statistical analyses presents complex challenges. This study introduces an innovative methodology for pinpointing regions of interest within brain MRI scans that are indicative of AD onset. Specifically, the proposed method tackles the challenges posed by irregular boundaries in the MRI scans and the interval‐censored nature of the data based on a flexible class of transformation models. By employing piecewise polynomial functions applied over triangulation, the brain images can be seamlessly integrated into the survival model and the region of interest can be subsequently identified within a regression‐based framework, enhancing the precision of AD prognosis. The efficacy of the proposed methodology is demonstrated using the ADNI dataset. A specific brain region has been identified as significantly associated with AD onset. In both axial and sagittal planes, the model with the selected region shows a smaller IBS (i.e., higher predictive accuracy) compared to the models without the imaging predictors and with all the imaging predictors. This finding offers valuable insights for clinical practitioners, especially in informing disease progression and risk stratification of patients.

The proposed method can be extended in the following ways. First, we utilize the brain MRI scans at baseline for risk prediction. However, the ADNI study has amassed longitudinal MRI scans with irregular revisiting time intervals, which presents an opportunity to enrich the predictive capability of the model. An advanced triangulation technique could be developed to capture features not only spatially across image pixels but also temporally across successive scans, potentially necessitating a three‐dimensional stochastic modeling framework for sparse functional data. Second, the proposed approach focuses on modeling two‐dimensional imaging data with irregular boundaries as predictors for survival outcomes. To further utilize the inherently three‐dimensional imaging data with irregular boundaries, the current method could be extended using, for example, the trivariate spline smoothing techniques recently proposed by Wang et al. [39]. Third, the proposed approach adopts a two‐stage estimation procedure. The initial stage involves approximating the image using Bernstein polynomials, followed by incorporating the approximated image into a survival model to perform group variable selection. While this method is computationally expedient, it is susceptible to the propagation of approximation errors from the first stage into the subsequent survival analysis. An alternative strategy could involve a joint modeling approach, wherein latent frailty terms are concurrently estimated, linking the imaging data directly with the latency component of the disease progression. Such methodological advancements would likely demand significantly increased computational resources.

## Conflicts of Interest

The authors declare no conflicts of interest.

## Data Availability

The R codes associated with this paper are openly available on GitHub: https://github.com/lcyjames/Roiico.

## Appendix A

### Conditional Expectations of the Latent Variables

Note that $E\left(A_{\mathrm{ij}}|A_{i}=0,O_{i},\theta^{\left(d\right)}\right)=0$ and $\left(A_{\mathrm{ij}}|A_{i}>0,O_{i},\theta^{\left(d\right)}\right)∼\text{Binomial}\left(A_{i},\mu_{\mathrm{ij}}/\sum_{l=1}^{J}\mu_{\mathrm{il}}\right)$. We have

$$\begin{array}{cc} \hat{E}\left(A_{\mathrm{ij}}\right)\equiv E\left(A_{\mathrm{ij}}|O_{i},\theta^{\left(d\right)}\right) & =E\left\{E\left(A_{\mathrm{ij}}|A_{i}>0,O_{i},\theta^{\left(d\right)}\right)|O_{i},\theta^{\left(d\right)}\right\}\\ & =E\left(\left.\frac{\mu_{\mathrm{ij}}A_{i}}{\sum_{l=1}^{J}\mu_{\mathrm{il}}}\right|A_{i}>0,O_{i},\theta^{\left(d\right)}\right)\\ & =\frac{\mu_{\mathrm{ij}}}{\sum_{l=1}^{J}\mu_{\mathrm{il}}}E\left(A_{i}|A_{i}>0,O_{i},\theta^{\left(d\right)}\right)\\ & =\frac{\mu_{\mathrm{ij}}}{\sum_{l=1}^{J}\;\mu_{\mathrm{il}}}E\left\{E\left(A_{i}|A_{i}>0,\zeta_{i},O_{i},\theta^{\left(d\right)}\right)|O_{i},\theta^{\left(d\right)}\right\}\\ & =\frac{\mu_{\mathrm{ij}}}{\sum_{l=1}^{J}\mu_{\mathrm{il}}}E\left\{\left.\frac{\zeta_{i}\sum_{l=1}^{J}\;\mu_{\mathrm{il}}}{1-e^{-\zeta_{i}\sum_{l=1}^{J}\;\mu_{\mathrm{il}}}}\right|O_{i},\theta^{\left(d\right)}\right\}\\ & =E\left\{\left.\frac{\zeta_{i}\mu_{\mathrm{ij}}}{1-e^{-\zeta_{i}\sum_{l=1}^{J}\mu_{\mathrm{il}}}}\right|O_{i},\theta^{\left(d\right)}\right\}, \end{array}$$

where the second last line refers to the mean of a zero‐truncated Poisson distribution. Similarly, we obtain

$$\hat{E}\left(B_{\mathrm{ij}}\right)\equiv E\left(B_{\mathrm{ij}}|O_{i},\theta^{\left(d\right)}\right)=E\left\{\left.\frac{\zeta_{i}\eta_{\mathrm{ij}}}{1-e^{-\zeta_{i}\sum_{l=1}^{J}\eta_{\mathrm{il}}}}\right|O_{i},\theta^{\left(d\right)}\right\}.$$

Thus, both $\hat{E}\left(A_{\mathrm{ij}}\right)$ and $\hat{E}\left(B_{\mathrm{ij}}\right)$ can be expressed analytically as the conditional expectation of a function of $\zeta_{i}$. Specifically, the conditional expectation of any function g of $\zeta_{i}$ given the observed data is

$$E\left\{g\left(\zeta_{i}\right)|O_{i}\right\}=\frac{\int g\left(\zeta \right)h_{i}\left(\zeta \right)\;d\zeta }{\int h_{i}\left(\zeta \right)\;d\zeta },$$

where

$$\begin{array}{ll} h_{i}\left(\zeta_{i}\right) & \equiv {\left\{1-e^{-\sum_{j=1}^{J}\zeta_{i}\omega_{j}I_{j}\left(R_{i}\right)e^{\beta^{T}Z_{i}+\gamma^{T}\xi_{i}}}\right\}}^{\Delta_{\mathrm{Li}}}\left\{e^{-\sum_{j=1}^{J}\zeta_{i}\omega_{j}I_{j}\left(L_{i}\right)e^{\beta^{T}Z_{i}+\gamma^{T}\xi_{i}}}\right.\\ & \;{\left.-e^{-\sum_{j=1}^{J}\zeta_{i}\omega_{j}I_{j}\left(R_{i}\right)e^{\beta^{T}Z_{i}+\gamma^{T}\xi_{i}}}\right\}}^{\Delta_{\mathrm{Ii}}}{\left\{e^{-\sum_{j=1}^{J}\zeta_{i}\omega_{j}I_{j}\left(L_{i}\right)e^{\beta^{T}Z_{i}+\gamma^{T}\xi_{i}}}\right\}}^{\Delta_{\mathrm{Ri}}}f_{\zeta }\left(\zeta_{i};\rho \right) \end{array}$$

is proportional to the conditional density of $\zeta_{i}$ given $O_{i}$. For notational simplicity below, let $S_{\mathrm{Li}}=\tilde{\Lambda }\left(L_{i}\right)e^{\beta^{T}Z_{i}+\gamma^{T}\xi_{i}}$ and $S_{\mathrm{Ri}}=\tilde{\Lambda }\left(R_{i}\right)e^{\beta^{T}Z_{i}+\gamma^{T}\xi_{i}}$. Note that $\hat{E}\left(A_{\mathrm{ij}}\right)$ is nonzero only if $\Delta_{\mathrm{Li}}=1$, in which

$$\hat{E}\left(A_{\mathrm{ij}}\right)=\frac{\mu_{\mathrm{ij}}}{e^{-G\left(S_{\mathrm{Li}};\rho \right)}-e^{-G\left(S_{\mathrm{Ri}};\rho \right)}}=\frac{\mu_{\mathrm{ij}}}{1-e^{-G\left(S_{\mathrm{Ri}};\rho \right)}}.$$

In the same vein, $\hat{E}\left(B_{\mathrm{ij}}\right)$ is nonzero only if $\Delta_{\mathrm{Ii}}=1$, in which

$$\hat{E}\left(B_{\mathrm{ij}}\right)=\frac{\eta_{\mathrm{ij}}e^{-G\left(S_{\mathrm{Li}};\rho \right)}G^{\prime }\left(S_{\mathrm{Li}};\rho \right)}{e^{-G\left(S_{\mathrm{Li}};\rho \right)}-e^{-G\left(S_{\mathrm{Ri}};\rho \right)}},$$

where $G^{\prime }$ denotes the first‐order derivative of G. Finally, we can also show that

$$\hat{E}\left(\zeta_{i}\right)=\frac{e^{-G\left(S_{\mathrm{Li}};\rho \right)}G^{\prime }\left(S_{\mathrm{Li}};\rho \right)-e^{-G\left(S_{\mathrm{Ri}};\rho \right)}G^{\prime }\left(S_{\mathrm{Ri}};\rho \right)}{e^{-G\left(S_{\mathrm{Li}};\rho \right)}-e^{-G\left(S_{\mathrm{Ri}};\rho \right)}}.$$

Therefore, all the conditional expectations in the E‐step have closed‐form expressions where numerical integration is not required, yielding a computationally efficient EM algorithm.
